# From Panoramic Radiographs to AI-Assisted Radiographic Periodontal Charting: Current Evidence and Future Perspectives

**DOI:** 10.3390/dj14090612

**Published:** 2026-09-20

**Authors:** Lluís Brunet-Llobet, Albert Ramírez-Rámiz, Judit Rabassa-Blanco, Pau Cahuana-Bartra, María Dolores Rocha-Eiroa, Elias Isaack Mashala, Jaume Miranda-Rius

**Affiliations:** 1 Department of Odontostomatology, Faculty of Medicine and Health Sciences, University of Barcelona, 08907 Barcelona, Spain; 2 Department of Dentistry, Hospital Sant Joan de Déu (HSJD), University of Barcelona, 08950 Barcelona, Spain; 3 Hospital Dentistry and Periodontal Medicine Research Group, Institut de Recerca Sant Joan de Déu (IRSJD), 08950 Barcelona, Spain; 4 Department of Orthopedic Surgery and Traumatology, Mount Meru Regional Referral Hospital, Arusha P.O. Box 3092, Tanzania

**Keywords:** artificial intelligence, periodontitis, panoramic radiography, periodontal charting, radiographic bone loss, radiographic attachment loss, clinical attachment loss, deep learning, diagnostic imaging, periodontal screening, dental radiology, precision dentistry

## Abstract

**Background:** Periodontal charting remains the gold standard for periodontal diagnosis but is time-consuming, operator-dependent, and not always feasible in routine clinical practice. In contrast, panoramic radiographs are widely available and increasingly amenable to automated analysis through artificial intelligence (AI). Recent AI systems have demonstrated high accuracy in the detection and quantification of periodontal bone loss and in radiographic disease staging. **Objective:** To review current evidence on AI applications in periodontal radiology and explore whether routinely acquired panoramic radiographs may support the development of AI-assisted radiographic periodontal charting. **Methods:** This narrative review summarizes recent evidence regarding AI-based periodontal and peri-implant radiographic assessment. The literature was identified through searches of PubMed, Scopus, and Google Scholar using combinations of terms related to artificial intelligence, periodontal disease, radiographic diagnosis, panoramic radiography, and peri-implantitis, with emphasis on studies relevant to AI-assisted periodontal assessment. **Results:** Recent deep learning architectures have demonstrated high diagnostic performance for periodontal bone loss detection, quantification, and disease staging. Current AI systems are capable of identifying radiographic manifestations of periodontal destruction and localising key anatomical landmarks, including the cemento-enamel junction and the alveolar bone crest. However, they remain unable to assess clinical parameters such as probing depth, bleeding on probing, suppuration, and tooth mobility. The review highlights the distinction between Clinical Attachment Level (CAL), Clinical Attachment Loss (CALoss), and radiographic measures of periodontal destruction. In this context, the concept of Radiographic Attachment Loss (RAL) is proposed as the radiographically measurable distance between the cemento-enamel junction and the alveolar bone crest, providing a conceptual estimate of periodontal support loss. **Discussion:** The principal novelty of this review is the proposal of AI-assisted radiographic periodontal charting as a clinically oriented framework for translating AI-derived radiographic information into meaningful periodontal assessment. Rather than replacing conventional periodontal charting, this approach aims to transform routinely acquired panoramic radiographs into a source of structured periodontal information that may support screening, risk stratification, longitudinal monitoring, and clinical decision-making. **Conclusions:** Artificial intelligence is unlikely to replace conventional periodontal examination. Nevertheless, AI-assisted radiographic periodontal charting may represent a realistic intermediate step between routine radiographic interpretation and comprehensive digital periodontal assessment. Future research should focus on validating AI-derived radiographic biomarkers, particularly Radiographic Attachment Loss (RAL), determining their clinical utility, and evaluating their potential contribution to periodontal screening, longitudinal monitoring and population-based oral health assessment.

## 1. Introduction

Periodontitis is a chronic multifactorial inflammatory disease characterized by the progressive destruction of tooth-supporting tissues and remains one of the leading causes of tooth loss worldwide. Contemporary classification systems developed by the American Academy of Periodontology (AAP) and the European Federation of Periodontology (EFP) emphasize the integration of clinical and radiographic findings to determine disease severity, complexity, and progression. Clinical attachment loss, radiographic bone loss, probing depth, furcation involvement, and tooth loss are among the key parameters used for diagnosis, staging, and grading of periodontitis [1,2,3,4,5].

Comprehensive periodontal diagnosis relies on periodontal charting, which remains the clinical gold standard for assessing periodontal health and disease. Through systematic examination, clinicians record probing pocket depth, bleeding on probing, gingival recession, suppuration, tooth mobility, furcation involvement, and clinical attachment level. Together, these measurements provide information regarding both historical periodontal destruction and current disease activity, supporting diagnosis, prognosis assessment, and treatment planning [6,7,8].

Despite its clinical value, periodontal charting presents several limitations. The procedure is time-consuming, requires trained personnel, and is subject to inter- and intraexaminer variability. Consequently, comprehensive periodontal charting is not always systematically performed in routine clinical practice, particularly in high-volume clinical settings [6,7,8].

Radiographic examination constitutes an essential complement to periodontal assessment. Periapical radiographs remain the two-dimensional reference standard for evaluating periodontal bone support owing to their high spatial resolution, whereas panoramic radiographs provide a comprehensive overview of both dental arches and are routinely acquired during dental examinations. Importantly, radiographic bone loss already plays a central role in contemporary periodontal classification systems. However, radiographic examinations only provide information on mineralized tissues and therefore cannot directly assess soft-tissue attachment or inflammatory activity [1,2,9].

Recent advances in artificial intelligence (AI), particularly deep learning, have transformed the analysis of dental images. Current AI systems are capable of automatically detecting periodontal bone loss, identifying anatomical landmarks, classifying disease severity, and supporting radiographic staging of periodontitis. Several studies and recent reviews have reported diagnostic performances approaching those of experienced clinicians, particularly when panoramic radiographs are used as the primary imaging modality [10,11,12,13,14,15,16,17,18].

Nevertheless, the role of AI in periodontology has largely been discussed in terms of whether it can replace conventional periodontal charting. This perspective may be overly restrictive. Rather than attempting to reproduce every component of a periodontal chart, AI may already be capable of automatically extracting clinically meaningful information from routinely acquired radiographic images [13,14,15,16,17].

In this context, the cemento-enamel junction (CEJ) and the alveolar bone crest represent two key anatomical landmarks. Their spatial relationship forms the basis of radiographic assessment of periodontal support and radiographic bone loss. Recent AI systems have demonstrated the feasibility of automatically identifying these landmarks on panoramic radiographs, thereby enabling the quantitative assessment of periodontal destruction and radiographic disease staging [19].

Beyond its diagnostic value, the increasing availability of panoramic radiographs in routine dental practice creates opportunities for the development of scalable AI-supported approaches to periodontal assessment. Unlike conventional periodontal charting, which requires dedicated clinical examination time, radiographic analysis can be retrospectively applied to existing imaging records, potentially facilitating screening and longitudinal follow-up in both clinical and public health settings. Building on these developments, it becomes important to distinguish between established clinical measures of periodontal support and radiographic indicators of periodontal destruction. The concept of Radiographic Attachment Loss (RAL) is explored in this review as a potential AI-derived radiographic biomarker of periodontal support loss [1,2,4,6,8,9].

Therefore, the aim of this review is to summarize current evidence regarding AI applications in periodontal radiology and to explore the emerging concept of AI-assisted radiographic periodontal charting, whereby artificial intelligence could transform routinely acquired panoramic radiographs into a source of structured and clinically meaningful periodontal information [9,13,14,15,16,17] (Table 1).

Conventional periodontal assessment relies on clinical examination and generates parameters such as Clinical Attachment Level (CAL) and Clinical Attachment Loss (CALoss). In contrast, artificial intelligence applied to panoramic radiographs may automatically identify the cemento-enamel junction (CEJ) and alveolar bone crest, enabling quantification of Radiographic Bone Loss (RBL) and estimation of the proposed concept of Radiographic Attachment Loss (RAL). AI-assisted radiographic periodontal charting is proposed as a complementary approach intended to support, rather than replace, conventional periodontal assessment.

## 2. Methods

This narrative review was based on a literature search conducted in PubMed, Scopus, and Google Scholar up to August 2026. Search terms included combinations of “artificial intelligence”, “deep learning”, “periodontitis”, “periodontal bone loss”, “panoramic radiograph”, “radiographic diagnosis”, “periodontal staging”, and “peri-implantitis”. Original studies, systematic reviews, meta-analyses, and narrative reviews were considered. Studies were selected on the basis of their relevance to AI-based periodontal and peri-implant radiographic assessment and their potential contribution to the development of the proposed concept of AI-assisted radiographic periodontal charting.

## 3. Results: Current Evidence on AI-Based Periodontal Radiographic Assessment

### 3.1. What Can and Cannot Be Derived from Panoramic Radiographs?

Conventional periodontal charting integrates clinical, biological, and radiographic information to provide a comprehensive assessment of periodontal status. However, not all periodontal parameters are equally accessible through radiographic imaging. Clinical variables such as probing pocket depth, bleeding on probing, suppuration, gingival inflammation, and tooth mobility require direct clinical examination and cannot be visualized on conventional radiographs. Consequently, these parameters remain beyond the capabilities of current image-based artificial intelligence systems [6,7,8,9].

In contrast, several parameters directly related to periodontal tissue destruction can be assessed radiographically. These include alveolar bone height, radiographic bone loss, infrabony defects, furcation involvement, peri-implant bone loss and the spatial relationship between the cemento-enamel junction (CEJ) and the alveolar bone crest. Importantly, these structures and measurements represent the primary targets of contemporary AI systems developed for periodontal image analysis [9,13,14,15,16,17].

Recent advances in deep learning have demonstrated a high degree of accuracy in detecting, segmenting, and quantifying many of these radiographic features. Consequently, AI is increasingly being explored as a tool capable of transforming routine radiographic examinations into sources of objective and reproducible periodontal information [13,14,15,16,17].

These observations suggest that the objective of AI-assisted periodontal assessment should not necessarily be the reproduction of a complete periodontal chart. Rather, artificial intelligence may be capable of automatically extracting clinically meaningful radiographic information that complements conventional periodontal examination and contributes to disease detection, staging and monitoring [9,13,14,15,16,17] (Table 2).

### 3.2. From CEJ Detection to Radiographic Attachment Loss

Among all radiographically identifiable structures, the cemento-enamel junction (CEJ) and the alveolar bone crest are arguably the most relevant anatomical landmarks for periodontal assessment. Their identification forms the basis of radiographic evaluation of periodontal support and underlies the quantification of radiographic bone loss. In contemporary periodontal classification systems, radiographic bone loss already plays a central role in periodontitis staging and grading [1,2,4,9].

Recent advances in artificial intelligence have demonstrated that these landmarks can be identified automatically with a high degree of accuracy. Xue et al. developed a deep learning framework capable of localizing the CEJ, alveolar bone crest, and root apex on panoramic radiographs, enabling automated calculation of the degree of alveolar bone loss and radiographic periodontitis staging [19].

**Table 2:** Comparison of conventional clinical periodontal parameters and AI-derived radiographic parameters.

Parameter	Conventional Periodontal Charting	Panoramic Radiograph	Current AI Assessment
Probing Pocket Depth (PPD)	Yes	No	No
Bleeding on Probing (BOP)	Yes	No	No
Suppuration	Yes	No	No
Gingival Recession	Yes	No	No
Tooth Mobility	Yes	No	No
Clinical Attachment Level (CAL)	Yes	No	No
Clinical Attachment Loss (CALoss)	Yes	No	No
Cemento-Enamel Junction (CEJ)	Reference landmark	Yes	Yes
Alveolar Bone Crest	No	Yes	Yes
Radiographic Bone Loss (RBL)	No	Yes	Yes
Disease Staging Support	Partial	Yes	Yes
Radiographic Attachment Loss (RAL)	No	Proposed	Potential

As summarized in Table 2, conventional periodontal assessment and AI-assisted radiographic assessment provide different but potentially complementary sources of periodontal information. While clinical examination generates parameters such as CAL and CALoss, AI analysis of panoramic radiographs may derive radiographic parameters including RBL and the proposed concept of RAL.

The ability to automatically identify the CEJ and alveolar bone crest is particularly relevant because these landmarks provide the anatomical basis for quantifying periodontal support loss visible on radiographic images. If AI systems can consistently and reproducibly detect both structures, automated calculation of the distance between them becomes feasible. This measurement may provide a quantitative representation of periodontal destruction and could be incorporated into structured radiographic assessment systems [9,19].

For this reason, the present review proposes the concept of Radiographic Attachment Loss (RAL), defined as the radiographically measurable distance between the CEJ and the alveolar bone crest. Unlike Clinical Attachment Level (CAL), which is measured clinically from the CEJ to the base of the periodontal pocket, and Clinical Attachment Loss (CALoss), which reflects cumulative loss of periodontal support assessed through clinical examination, RAL is derived exclusively from radiographic landmarks [6,8,9].

Importantly, RAL should not be considered equivalent to CAL or CALoss. Rather, it should be regarded as a radiographic estimate of periodontal support loss that complements clinical assessment. Although it cannot provide information regarding probing depth, soft tissue attachment, or inflammatory activity, RAL may represent a clinically meaningful radiographic biomarker of periodontal destruction that can potentially be standardized and automated through AI-based image analysis [9].

### 3.3. Current AI Applications in Periodontology and Implant Dentistry

The detection and quantification of periodontal bone loss represent the most extensively investigated applications of AI in periodontology. Recent deep learning architectures, including convolutional neural networks, U-Net variants, YOLO-based frameworks, and transformer-based models, have demonstrated high diagnostic performance for bone loss detection, disease classification, and periodontitis staging on panoramic radiographs. Reported diagnostic accuracies generally range from approximately 77% to over 95%, depending on the diagnostic task, dataset characteristics, and study design [19,20,21,22,23,24,25,26,27] (Table 3).

Beyond simple detection of periodontal destruction, current AI systems are increasingly capable of performing more complex diagnostic tasks, including automated localization of anatomical landmarks, disease severity classification, and radiographic staging of periodontitis. These developments suggest that AI is evolving from a purely image-analysis tool towards a more comprehensive diagnostic support system [19,21,23,26].

The application of AI to peri-implant diseases remains comparatively limited but has produced encouraging results. Recent studies have demonstrated promising performance for the detection of peri-implant bone defects and the automated quantification of marginal bone loss, indicating that AI applications may extend beyond periodontitis and contribute to the assessment of peri-implant tissue breakdown [18,25].

Despite these advances, none of the currently available AI systems are capable of evaluating essential clinical parameters such as probing pocket depth, bleeding on probing, suppuration, gingival recession, or tooth mobility. Consequently, current evidence supports AI as a valuable adjunctive tool rather than a replacement for conventional periodontal charting [6,8,9,14,15,16,17].

Table 3 illustrates that current AI applications in periodontal and peri-implant radiology are primarily focused on landmark detection, periodontal diagnosis, radiographic staging, and bone loss quantification.

## 4. Discussion

### 4.1. Clinical and Radiographic Measures of Periodontal Support

One of the most important conceptual considerations arising from the current literature concerns the distinction between clinical and radiographic measures of periodontal support. While contemporary AI systems have demonstrated increasing capability in the analysis of radiographic periodontal destruction, their outputs should not be interpreted as direct equivalents of established clinical periodontal parameters [1,2,6,10].

Clinical Attachment Level (CAL) remains one of the most relevant clinical indicators of periodontal attachment status and is defined as the distance from the cemento-enamel junction (CEJ) to the base of the periodontal pocket. Likewise, Clinical Attachment Loss (CALoss) represents the cumulative loss of periodontal support and continues to play a central role in the diagnosis and staging of periodontitis within the current AAP/EFP classification system [1,2,6,8].

By contrast, radiographic examinations provide information exclusively on mineralized structures. Consequently, AI systems analysing radiographic images cannot directly measure CAL or CALoss. However, they are capable of identifying key anatomical landmarks, particularly the CEJ and the alveolar bone crest, which form the basis of radiographic assessment of periodontal support [9].

The feasibility of automatically identifying these landmarks has already been demonstrated by recent deep learning systems. Xue et al. developed an AI framework capable of localising the CEJ, alveolar bone crest, and root apex on panoramic radiographs in order to calculate alveolar bone loss degree and support automated periodontitis staging. These findings suggest that landmark-based radiographic assessment is technically achievable and may serve as the foundation for more advanced clinical applications [19].

Based on these observations, the concept of Radiographic Attachment Loss (RAL) is proposed as the radiographically measurable distance between the CEJ and the alveolar bone crest. Importantly, RAL should not be considered equivalent to CAL or CALoss. Rather, it should be regarded as a radiographic estimate of periodontal support loss derived exclusively from mineralised tissue evaluation [9].

Although RAL cannot replace clinical attachment measurements because it does not incorporate information regarding soft tissues or inflammatory activity, it may provide a standardised and clinically meaningful radiographic biomarker of periodontal destruction. Future prospective studies should evaluate the relationship between RAL, Radiographic Bone Loss (RBL), CAL and CALoss in order to determine whether AI-derived measurements can contribute to more precise and standardised periodontal assessment [1,2,8,10].

### 4.2. AI-Assisted Radiographic Periodontal Charting: A New Conceptual Framework

Traditionally, research on artificial intelligence in periodontology has focused on determining whether AI can replace periodontal charting. However, the evidence reviewed here suggests that this question may be overly restrictive.

The novelty of the present review does not lie in demonstrating that artificial intelligence can detect periodontal bone loss, as this capability has already been consistently reported in the literature. Rather, it arises from the proposal of a clinically oriented framework aimed at translating these technological advances into meaningful periodontal assessment tools. Instead of focusing on marginal improvements in diagnostic accuracy, future developments may depend on the identification of clinically relevant ways to integrate AI-derived radiographic information into periodontal decision-making [13,14,15,16,17,18,19,21,23].

Rather than attempting to reproduce every component of a periodontal chart, artificial intelligence could be used to automatically extract periodontal parameters that are visible on radiographic examinations. Current evidence indicates that AI systems can already identify alveolar bone loss, classify disease severity, detect peri-implant bone defects, and support radiographic staging with a high degree of accuracy [19,20,21,23,26,27,28].

The proposed framework is primarily intended to provide structured tooth-level radiographic information derived from panoramic radiographs. At a minimum, the proposed output would include radiographic bone loss (RBL), Radiographic Attachment Loss (RAL), and disease severity estimates, with the potential to support the generation of standardized radiographic periodontal reports.

If future AI systems achieve robust and reproducible identification of the CEJ and alveolar bone crest across different clinical scenarios, automated calculation of Radiographic Attachment Loss may become feasible. Such information could be incorporated into a structured radiographic report containing tooth-specific measurements of periodontal support, radiographic bone loss, furcation involvement, disease severity, and potential risk areas [9,19].

Accordingly, this review proposes the concept of AI-assisted radiographic periodontal charting, whereby artificial intelligence is not intended to replace conventional periodontal examination but rather to transform routinely acquired panoramic radiographs into a source of structured, quantitative, and clinically meaningful periodontal information. This framework may support screening, longitudinal monitoring, disease staging, patient communication, and clinical decision-making while complementing conventional periodontal assessment [9,14,15,16,17,19].

Under this perspective, the key challenge for future research may not be to achieve incremental improvements in algorithmic accuracy, but rather to determine how AI-derived radiographic parameters can be integrated into clinically relevant workflows. The transition from automated radiographic measurements to clinically meaningful periodontal assessment may represent the next step in the evolution of artificial intelligence in periodontology [19].

### 4.3. Clinical Implications and Future Directions

From a clinical perspective, one of the most attractive aspects of AI-assisted radiographic periodontal charting is its favourable cost–benefit profile. Panoramic radiographs are already routinely obtained in many dental practices, hospitals, and screening settings. Therefore, automatic extraction of periodontal information would not require additional radiation exposure, additional chairside time or additional diagnostic procedures. The clinician would simply obtain more clinically useful information from an image that is already available [9].

Importantly, a clinically useful system does not need to reproduce the entire periodontal chart to provide significant value. Even if AI systems were capable of generating only part of the information included in conventional charting, such as radiographic bone loss, Radiographic Attachment Loss, disease severity estimates, and identification of high-risk teeth, the resulting information could substantially improve periodontal screening and diagnostic workflows [9,14,15,16,17,19].

This approach may be particularly relevant in public health programs, epidemiological studies, general dental practice, and healthcare environments where comprehensive periodontal charting is not always feasible. In these settings, AI-assisted interpretation of routinely acquired panoramic radiographs could facilitate the early identification of patients requiring further periodontal assessment.

As a screening tool, AI-assisted radiographic periodontal charting may also support the identification of patients who could benefit from a comprehensive periodontal examination. The detection of predefined patterns of Radiographic Attachment Loss (RAL) affecting multiple teeth or anatomical regions could potentially trigger recommendations for periodontal evaluation and, when appropriate, referral to a periodontal specialist. However, the clinical validity and diagnostic performance of such screening strategies require further investigation.

Several authors have suggested that the next generation of AI platforms should integrate radiographic findings with periodontal probing measurements, bleeding scores, clinical attachment loss, behavioural risk factors, systemic conditions, and biological biomarkers. Such multimodal systems could overcome some of the intrinsic limitations of image-based diagnosis and provide a more comprehensive assessment of periodontal status [20,22].

In addition, comparison of panoramic radiographs acquired at different time points may help clinicians visualize and monitor the radiographic progression of periodontitis over time, thereby supporting longitudinal patient follow-up. Automated longitudinal assessment of Radiographic Attachment Loss (RAL) derived from serial panoramic radiographs may facilitate the detection of subtle changes in periodontal support, helping clinicians identify disease progression patterns that might otherwise remain unnoticed during routine radiographic interpretation. This approach may also have important educational value, as visual comparison of serial radiographic examinations could help patients better understand the progression of their periodontal condition, potentially improving disease awareness, treatment adherence, and long-term periodontal maintenance.

Most currently published AI models remain research-oriented systems and have not yet undergone sufficient external validation for routine clinical implementation. Panoramic radiographs are also affected by intrinsic limitations, including image distortion, magnification, patient positioning errors, anatomical superimposition, and variability in image acquisition protocols. These factors may influence the visibility of anatomical landmarks and should be considered when interpreting AI-derived radiographic measurements, particularly in longitudinal assessments.

Nevertheless, important limitations remain. The proposed concept of Radiographic Attachment Loss (RAL) requires clinical validation, and its relationship with established periodontal parameters remains to be clarified. Furthermore, AI-assisted radiographic periodontal charting should be regarded as a complementary rather than substitute approach, since essential parameters such as probing depth, bleeding on probing, suppuration, and tooth mobility remain inaccessible to image-based systems [6,8,9].

Future research should therefore focus on validating AI-derived radiographic biomarkers, determining their relationship with conventional periodontal measurements, and exploring their integration into clinical decision-support systems. Such advances may help bridge the gap between routine radiographic interpretation and comprehensive digital periodontal diagnostics [19,21,23].

## 5. Conclusions

Artificial intelligence has emerged as a promising tool for the analysis of periodontal radiographic images and is increasingly capable of detecting periodontal bone loss, identifying anatomical landmarks, and supporting radiographic disease staging from routine panoramic radiographs. However, AI cannot replace conventional periodontal charting because essential clinical parameters, including probing depth, bleeding on probing, suppuration, and tooth mobility, remain inaccessible to image-based systems. Therefore, AI should currently be regarded as a complementary diagnostic tool rather than a substitute for comprehensive periodontal assessment.

The principal contribution of this review is the proposal of Radiographic Attachment Loss (RAL) and AI-assisted radiographic periodontal charting as a conceptual framework for translating AI-derived radiographic information into clinically meaningful periodontal assessment. Future research should focus on validating AI-derived radiographic biomarkers, particularly Radiographic Attachment Loss (RAL), and determining whether AI-assisted radiographic periodontal charting can contribute to periodontal screening, longitudinal monitoring, and population-based oral health assessment. The increasing availability of longitudinal radiographic datasets may further facilitate the development of AI-supported tools for monitoring periodontal health over time and to improve patient engagement in periodontal care.

## Figures and Tables

**Table 1:** Conceptual framework of AI-assisted radiographic periodontal charting. * RAL: proposed conceptual radiographic parameter requiring future clinical validation.

Clinical Pathway	AI Radiographic Pathway
Conventional Periodontal Assessment	AI-Assisted Radiographic Assessment
Clinical Examination:	Panoramic Radiograph + AI:
Probing Pocket Depth (PPD)	Automatic Landmark Detection
Bleeding on Probing (BOP)	Cemento-Enamel Junction (CEJ)
Gingival Recession	Alveolar Bone Crest
Suppuration	Automated Radiographic Assessment:
Tooth Mobility	Radiographic Bone Loss (RBL)
Clinical Attachment Level (CAL)	Radiographic Attachment Loss (RAL) *
Clinical Attachment Loss (CALoss)	Disease Severity Estimation
↓	↓
Periodontal Charting	AI-Assisted Radiographic Periodontal Charting

**Table 3:** Representative studies on AI-based periodontal and peri-implant radiographic assessment.

Study	Imaging Modality	AI Task	Main Output	Validation
Xue et al. 2024 [19]	Panoramic radiograph	Landmark detection, staging	CEJ, bone crest, staging	Internal
Li et al. 2025 [20]	Panoramic radiograph	Periodontitis detection	Stage II-IV diagnosis	Multicentre
Le et al. 2026 [22]	Panoramic radiograph	Diagnosis and staging	Automated classification	Internal
Adnan et al. 2026 [25]	Panoramic radiograph	Bone loss quantification	Marginal bone loss	Internal
Jiang et al. 2022 [26]	Panoramic radiograph	Staging	Bone loss stages	Internal
Xia et al. 2026 [27]	Panoramic radiograph	Infrabony defect detection	Regenerative defects	Multicentre

## Data Availability

The datasets generated and analyzed during the current study are available from the corresponding author upon reasonable request.
